# Efficacy of Nutrition and WASH/Malaria Educational Community-Based Interventions in Reducing Anemia in Preschool Children from Bengo, Angola: Study Protocol of a Randomized Controlled Trial

**DOI:** 10.3390/ijerph16030466

**Published:** 2019-02-05

**Authors:** Cláudia Fançony, Ânia Soares, João Lavinha, Henrique Barros, Miguel Brito

**Affiliations:** 1 Health Research Center of Angola (CISA), Caxito, Estação Central de Luanda, Apartado IV n°5547, Luanda, Angola; ania.soares@cisacaxito.org (Â.S.); miguel.brito@cisacaxito.org (M.B.); 2 Institute of Public Health, University of Porto, 4050-091 Porto, Portugal; hbarros@med.up.pt; 3 Department of Human Genetics, National Health Institute Doctor Ricardo Jorge, 1649-016 Lisbon, Portugal; Joao.lavinha@insa.min-saude.pt; 4 BioISI, Faculty of Science, University of Lisbon, 1749-016 Lisbon, Portugal; 5 Health and Technology Research Center, Escola Superior de Tecniologia da Saúde de Lisboa, Instituto Politécnico de Lisboa, 1990-096 Lisbon, Portugal

**Keywords:** Anemia, malnutrition, infections, educational interventions, nutrition, WASH/Malaria

## Abstract

Angola reports one of the highest infant mortality rates in the world, and anemia represents one of its important causes. Recent studies, in under-five children from the Bengo province of Angola, described high prevalence’s, suggesting malaria, undernutrition and urogenital schistosomiasis as important contributors for the occurrence and spatial variations of anemia. Educational community-based interventions, either in Nutrition and Water, Sanitation, Hygiene and Malaria are recommended to correct anemia. Herein, we designed a cluster-randomized controlled trial to study the efficacy of two educational-plus-therapeutic interventions in the reduction of anemia: one in nutrition and the other in WASH/Malaria. Socioeconomic, nutritional, anthropometric, parasitological and biochemical data will be collected from all willing-to-participate children, aging under four and resident in the Health Research Center of Angola study area. Considering the multifactorial causes of this condition, determining the efficacy of both interventions might help documenting weaknesses and opportunities for planning integrated strategies to reduce anemia.

## 1. Background

Anemia, especially severe anemia, is associated with increased child mortality rates [1]. Moreover, limitations in physical and cognitive development, leading to an increase in academic failure and susceptibility to infectious diseases, are also of great concern [1,2,3,4]. Regional studies conducted in the Dande municipality (Bengo province) reported a 57% prevalence of anemia in under-5 children, indicative of a severe public health problem [5]. In this setting, in addition to the expected association with undernutrition (responsible for nearly 13% of anemia cases), clusters of high risk of anemia were found to overlap with *Plasmodium falciparum* and *Schistosoma haematobium* risk zones (associated with 16% and 10% of the cases, respectively) [5,6].

Around half of anemia cases are estimated to result from Iron Deficiency (ID) [7]. However, in malaria endemic countries, routine iron supplementation remains controversial and is only undertaken with caution, as the practice has been associated with both an increased incidence and severity of malaria, especially in non-iron-deficient anemic people [8,9,10,11,12]. Additionally, single nutrient deficiencies are rare, as insufficient ingestion of other hematopoietic micronutrients may occur and cause nutritional anemia. On the other hand, the high prevalence, intensity, reinfection and incidence rates of anemia-related infections can also contribute to iron deficiency anemia, potentially refractory to iron supplementation [2,5,13,14,15,16].

Integrating therapeutic (deworming) and preventive (either food-based or WASH/malaria education) strategies can simultaneously treat infections and increase hematopoietic micronutrient intake or reduce disease transmission, which could result in the reduction of malnutrition and anemia [2,14,17,18,19,20,21,22,23,24,25,26,27,28,29,30,31,32]. Several comprehensive intervention strategies have correspondingly been tested. However, they frequently evaluate the impact of education on nutrition or on infectious etiologies (whether or not combined with drug therapy) separately [17,18,19,20,21,22,23,24,25,26,27,28,29,31]. Thus, differences in their designs and methodologies hinder the comparison and evaluation of results, rendering it difficult to define the best approach to reduce anemia. For instance, differences on the target population (adult or children), dimensions of the learning package (e.g., for sanitation: stool disposal, water quality or supply), the delivers (community promoters/volunteers, teachers, local health workers, community leaders), the place where education occurs (at health center or at the communities), the number of intervention contacts, type of contacts (group meetings and/or individual contacts), duration of the intervention/observation and mainly the combination with other strategies (micronutrient supplementation, construction of latrines and hand-washing mechanisms, etc.) can be observed [17,18,19,20,21,22,23,24,25,26,27,28,29,31]. Furthermore, one of the most inclusive studies was published recently by Humphrey et al. 2019, and investigated the effect of two WASH interventions, either alone or combined with improved Infant and Young Child Feeding (IYCF), concluding that education in nutrition reduce anemia and stunting, but adding WASH to the intervention had no major improvements on those effects [33]. Similar results were reported by Null et al. 2018 within a cluster-randomized trial using interventive groups with several WASH and nutrition combinations [34]. Neither study investigated the effect of combining deworming, nor was the impact of malaria preventive actions included, and as far as we know, there are no published protocols or results of such studies implemented in Angola.

### 1.1. Aim

This paper describes the design of a cluster-randomized controlled trial that aims to compare the efficacy of two complex, community-based interventions: (1) nutritional education in the reduction of nutritional anemia, and (2) WASH/malaria education in the reduction of anemia caused by infection, both combined with a test-and-treat therapeutic approach.

### 1.2. Hypotheses under Study

(1) The nutrition arm assumes that the test-and-treat approach will clear infections and that education in nutrition will improve the knowledge and awareness of caretakers as regards the causes, prevention and treatment of nutritional anemia, which in turn may result in behavioral changes that reduce the prevalence of inadequate breastfeeding and IYCF. This could in turn improve dietary diversity, by increasing the consumption of erythropoietic-rich foods (iron, vitamin B12, vitamin A and folate) and enhancers of iron absorption (vitamin C), and decreasing the inadequate consumption of iron absorption inhibitors (polyphenols, phytates, calcium, etcetera)) [24,35,36], leading ultimately to a decrease in the prevalence of nutritional anemia.

(2) The WASH/malaria arm assumes both that the test-and-treat approach clears infections, and that education in appropriate water, sanitation, personal hygiene and malaria prevention practices improves the knowledge of caretakers regarding the causes and preventive measures against infections, which in turn would lead to behavioral changes, and consequently to reduced prevalence, intensity, incidence and re-infection rates of those diseases [31,37,38,39,40]. Thus, considering that parasitic infections are important causes of anemia within this setting, this intervention would consequently decrease the occurrence of infection-related anemia.

In our study, the intervention in the control group targets the treatment of infections, the intervention in the nutrition arm targets nutritional anemia and nutrition-related causes, and the WASH/malaria intervention focuses on infections and infectious-related anemia. Furthermore, it should be considered that non-malarial infections can also cause anemia through nutritional deficiencies, by causing blunted intestinal villi (which lead to impaired micronutrient absorption) [41]. Thus, by trying to prevent anemia through these 3 possible pathways, we can simultaneously investigate (1) whether combining education in nutrition with a test-and-treat approach could reduce nutritional anemia more than only treating intestinal/urogenital and malaria infections; (2) whether combining education in WASH/Malaria with a test-and-treat approach could reduce nutritional anemia more than only treating intestinal/urogenital and malaria infections; and 3) which of the educational interventions can prevent nutritional anemia the most.

## 2. Materials and Methods

### 2.1. Study Setting

This study targets hamlets with functional primary health care facilities within CISA’s Dande HDSS study area [42]. The surveyed area is located in the Bengo Province (Dande municipality), and includes all the hamlets in the communes of Úcua, Caxito, Mabubas, as well as some hamlets in Kikabo and Barra do Dande [42]. Within this area, between 2010 and 2014, the total resident population varied between 63,081 and 58,645, with the percentage of under-5 children ranging from 17.7% to 13.9%. Additionally, the fertility rate varied between 4.8 and 3.7 births per woman, with the crude birth rate ranging from 40.4 to 24.2 live births/year/1000 people and the under-5 mortality rate fluctuating between 92.1 and 60.1 per 1000 live births [42]. As the entire population of Kikabo and Barra do Dande are not fully surveyed by the Dande HDSS, they will not be included in this study. Although 15 hamlets with health facilities are located within this area, only those providing daily primary care practices will be included in this study [43].

### 2.2. Study Design

#### Baseline Assessment (Pre-Intervention Evaluation)

There were two qualitative pilot studies: (1) to characterize mother-to-child nutritional practices (as a basis to structure the questionnaire) and (2) to design a locally adequate Food Photography Atlas (FPA) for children U5 (for the 24 h recall evaluation). The first study was conducted between August and September of 2014 and enrolled 808 children aging 0–59 months and their caretakers in a two-stage cluster sampling strategy. Using a Portuguese translated questionnaire, adapted from ProPAN 14, we collected data on children’s breastfeeding, feeding practices and detailed food consumption in the previous 24 h [44]. As a result, data regarding the commonly consumed food was collected and a preliminary FPA S was produced. Following this, the second study, aiming at validating the previously mentioned FPA and to allow for the collection of age and context-specific food portion and nutrient ingestion among preschool children from the Bengo province, was conducted between February and March 2015. This was a convenient cross-sectional study that enrolled 75 primary caretaker residents in the Mabungo and Riceno hamlets within CISA’s study area. In sum, dishes were prepared according to local recipes, food photographs were produced, tested and selected, portion sizes were estimated and validated, and the reproducibility of this tool was evaluated. These results are being published elsewhere.

Using these tools, a pre-intervention evaluation, aiming to characterize the study population regarding anemia and its etiologic profile (by specific age groups (children aged under 6 months, between 6 and 23 months and between 24 and 36 months), will be performed.

### 2.3. Participants

All under-4 children and their mothers/caretakers, resident in the selected hamlets, will be considered eligible and invited to participate. This age group was selected because children are expected to have limited mobility, to receive higher parental attention and to have their exposure to contaminated environments more controllable than school aged children. Children will be subject to exclusion whenever having recently received blood transfusions, report adverse reactions to albendazole and/or to praziquantel, and when their caretakers do not commit to completing both the evaluation and the educational moments of the study. Only children with pre-intervention evaluation will be allocated to the Randomized Controlled Trial (RCT).

### 2.4. Training for Evaluation Moments

At the beginning of the study, 6 field workers and 2 nursing technicians will be selected from the study area and trained. The one-week training will incorporate: (1) an introduction to the research questions, goals and study design; (2) basic concepts regarding the diseases studied; (3) methodologies for data collection (structured interviews, anthropometric evaluations, recognition of signs and symptoms of micronutrient deficiency, measurement of temperature); and (4) communication skills and the collection/delivery of information. Additionally, nursing technicians will undergo 3-day training on: (1) the treatment and referral protocol adopted in this study; (2) best practices for drug administration in young children; and (3) the management of domiciliary and health unit treatments. Blood, urine and stool samples will be collected before their processing by experienced CISA resident laboratory technicians. A field work simulation class will be conducted with all personnel involved in this survey at the end of all training sessions.

### 2.5. Recruitment of Participants

A house-to-house recruitment team will identify the children eligible in each hamlet, explaining the importance of the study both to local authorities and caretakers, delivering stool and urine containers and explaining the best practices for sample collection. This team will recruit up to 45 children per day, and orient them to attend the next evaluation at the hamlet health post. Those evaluations will be carried out in each hamlet until all eligible willing-to-participate families have been notified.

### 2.6. Baseline Data Collection and Sample Storage

A structured questionnaire is to be administered to mothers or other primary caretakers to collect information on identification, breastfeeding, complementary feeding practices and health care practices, supplementation and vaccination history, WASH and malaria practices. Weight and height, collected using electronic or platform pediatric scales and measuring harness or adult stadiometer, will be used to calculate anthropometric indices for the diagnosis of undernutrition in children and mothers according to WHO standards [45,46]. Peripheral blood will be collected in accordance with WHO good phlebotomy practices [47]. For biochemical analyses, blood will be collected in 1.1ml Z-Gel microvette tubes, centrifuged for the separation of the serum and stored at −20 °C and blood for molecular analysis will be stored in air-dried filter paper until processing. Mothers/caretakers will be instructed to collect a stool sample on the day before evaluation and to collect urine in the morning of evaluation day. Urine samples are to be conserved in formalin (10%) to prevent larvae eclosion.

### 2.7. Sample Processing

Stool, urine and blood will be analyzed parasitologically. Blood protozoa (*Plasmodium falciparum* and *Plasmodium vivax*) will be determined by the Rapid diagnostic test (*SD BIOLINE* Malaria Ag P.f/P.v (HRP-2/pLDH, Standard Diagnostics, INC, Suwon City, Korea))*,* intestinal helminths (*Ascaris lumbricoides, Trichuris trichiura*, *Hymenolepis nana* and *Strongyloide stercoralis*) determined through fecal smear according to the Katokatz technique, intestinal protozoa (*Giardia lamblia* and *Entamoeba hystolitica/dispar*) evaluated through fecal centrifugal-sedimentation (using Parasitrap® System, Biosepar, Mühldorf, Germany) and urogenital helminth (*Schistosoma haematobium*) determined by urine filtration [48,49]. Biochemical analyses will comprise quantifying hemoglobin directly from peripheral blood (using Hemocue ® Hb 301 System, Angelholm, Sweden), serum ferritin, C-reactive protein and zinc through kits from Quimica Clínica Aplicada (Quimica Clínica Aplicada S.A., Tarragona, Spain) and an automated analyzer (BT1500, Biotecnica Instruments S.p.A, Rome, Italy), serum retinol through high-performance liquid chromatography with a protodiode-array detection, and vitamin B12 and folic acid by chemiluminescence immunoassay (using VITROS® Immunodiagnostic kits, Ortho Clinical Diagnostics, Inc. Bridgend, UK) [50,51,52,53]. Molecular analysis will include DNA extraction (by Chelex method), screening for sickle cell trait and disease (by PCR) and G6PD deficiency (by rtPCR) [54,55]. Malaria diagnosis will take place on site, and stool/urine samples will be analyzed within 24/48 h of collection (depending on the day of the week). The laboratory analyses will be performed by trained technicians and 25% of tests subject to confirmation by experienced laboratory supervisors.

### 2.8. Drug Therapy

Children diagnosed with *Plasmodium falciparum* malaria, urogenital schistosomiasis and/or intestinal parasites, will receive treatment, respectively with artemether/lumefantrine (20/120, plus paracetamol (15 mg/kg/dose for temperatures above 38 °C)), albendazol (400 mg/single dose for *Ascaris lumbricoides*, *Trichuris trichiura* and hookworms) and praziquantel (40 mg/kg for *Schistosoma haematobium* and *Schistosoma mansoni* (splited into two intakes, 6 h apart) and 25 mg/kg for *Hymenolepis nana*), according to the national therapeutic guidelines and specialist bibliography [56,57,58,59]. *Plasmodium falciparum*-infected children are to be treated on the evaluation site (unless signs of severe malaria are observed) and all albendazole treatments (except for children aged younger than one) will be administered in the household by nurse technicians. The remaining infected children will be treated at specific consultations with CISA’s pediatrician at the health centers. Furthermore, any children diagnosed with sickle cell disease will be referred to the Anemia Patient Follow-up Consultation, held at the Bengo General Hospital. Drugs will be provided by CISA through the entire duration of the study.

## 3. Cluster-Randomized Controlled Trial

### 3.1. Sampling Strategy

In this study, the sampling strategy chosen was a non-probabilistic (convenient) sampling. In sum, we aimed to include (through a census approach) all eligible children (under the age of 4) resident in administratively and geographically isolated hamlets with functional health posts, in turn located within the CISA’s HDSS study area (considered as cluster units). We chose hamlets with functional health posts due to the greater facility in mobilizing the population and corresponding logistical advantages. On the other hand, the census approach (within those clusters) was adopted because variations in the density of eligible children, estimated according to data from 24-10-2014 extracted from CISA’s HDSS database, was expected, and the real density in each cluster was needed.

### 3.2. Randomization

The hamlets will be considered as cluster units. For randomization, the names of the hamlets will be written down on pieces of paper and placed in a bag and then successively removed. The first two papers to be removed will be attributed to the Nutrition arm, the following two to the WASH/Malaria arm and the next pair to the control with this procedure repeated until there are no papers left in the bag. This process will be carried out by the two CISA researchers coordinating the study.

### 3.3. Blinding

This will be an unblinded study.

### 3.4. Training of Providers

Field and nursing technicians involved in the evaluation (baseline and follow up waves) will be assigned to the different intervention arms, being responsible for the counselling sessions (monitoring, encouraging and continuously promoting behavioral change), under the direction of a supervisor. Their previous training will be reinforced with theoretical and practical sessions on (1) the core aspects of the target disease and conditions (specifically, “What is the disease/condition”, “What are the signs and symptoms”, “What is causing them”, “What can mothers do to prevent them”), (2) counselling techniques (evaluating the emotional state of the caretaker and adapting counselling techniques to the emotional state), (3) household environment and risk behavior evaluation, according to the respective interventional arm they belong to. Within each intervention arm, they will perform one cycle of three-monthly visits to a group of households before then moving onto another group of families.

Following the first training stage, refresher sessions will occur before the follow up evaluation moments and before every domiciliary personalized counselling. At the beginning and at the end of each training stage, technicians will undertake a written evaluation test and a score will be generated. Additionally, 20% of all counselling sessions (per technician) will be subject to evaluation by a supervisor, who will similarly produce a counselling score for each trainee.

### 3.5. Training of Receivers

The receivers, i.e., the targets for the behavior change interventions, will be mothers and/or primary caretakers of the children studied. After the baseline assessment, the mothers in each intervention arms will be trained according to the respective “targets within the educational package” described in Table 1. The objective is to empower mothers with knowledge of basic principles regarding: (1) anemia and its nutrition-related etiologies and (2) anemia and its infections-related etiologies (in particular, what are the signs and symptoms? How did the child become at risk? How can mothers prevent this and what they should do whenever their children fall sick?) and to stimulate inadequate mother-to-child behavior changes. Technicians will document pre-and post-intervention alterations in the household environment and mother-to-child practices (according to the process indicators described in Table 2).

### 3.6. Recruitment and Promotion of Adherence

To relocate families, field technicians will perform a house-to-house invitation strategy, using Dande’s HDSS household identification system, reference points and family phone numbers. To increase adherence and promote participant retention, the hamlet coordinators and/or traditional authorities will be invited to be present at all phases of the study. Furthermore, incentive kits will be distributed to all children during the follow up moments, namely a nutritional kit (water and yogurt) and a WASH/Malaria kit (soap and lye).

### 3.7. Educational Package for Receivers

Families/caretakers allocated to the interventions are to receive six personalized counselling sessions at their homes and will be invited to participate in six community workshops for practice lessons. The overall treatment given in each arm, throughout the duration is described in Table 1.

Illustrative materials, adapted from UNICEF, will be used for these counselling sessions, as well as posters highlighting key health messages [60,61].

The practical cooking lessons within the nutrition study arm will aim at reinforcing the adoption of a diet with a Minimum Dietary Diversity and Minimum Meal Frequency and at increasing iron, folic acid, vitamin A- and B12-rich food ingestion [62]. Accordingly, after evaluating the ingredients available at the local market, aliments within the food groups of (1) grains, roots and tubers, (2) legumes and nuts, (3) dairy products, (4) flesh foods, (5) eggs, (6) vitamin A-rich fruits and vegetables, and (7) other fruits and vegetables will be identified, and easy, affordable and pleasant-to-the-palate recipes will be developed. Within those groups, food sources of heme-iron (red meat and poultry) and non-heme iron (specifically green leaves), will receive major attention [63,64,65,66,67]. Data collected at the qualitative study mentioned above, aiming at assessing the main feeding practices of infants and young children, will be used to assist this purpose. Classes will comprehend: (1) discussing the nutritional value of local foods, the effect of processing them on their nutrient value, (2) identifying the most common foodborne parasites, (3) demonstrating the fish-and-meat dishes to be used in the meals, (4) demonstrating combinations to be used at breakfast and lunch, (5) tasting and scoring recipes and (6) distributing illustrative recipe notebooks for practice.

The practical sessions within the WASH/malaria intervention arm will aim at preventing malaria, schistosomiasis and STH in children, particularly decreasing the transmission rate and consequently lowering their incidence and reinfection rates. Activities within the community groups will include: (1) identifying local points of possible STH contamination and children’s risk behaviors, (2) visiting target households to identify mosquito indoor resting and outdoor breeding sites, (3) constructing household washing-hand mechanisms, (4) correctly mounting of bed-nets and (5) running child hand-washing classes.

## 4. Data Collection

### Educational Interventions

Caretakers participating in the intervention arms will have a monitoring file (consisting of six structured questionnaires (one for each monthly visit), applied by the technician to document behavioral alterations at every counselling visit and to record attendance to the community group sessions. Those educational interventions will be intercalated with cross-sectional evaluations. Those moments will have the same design as the baseline assessment, occurring respectively six and twelve months after the beginning of the study.

Data on the primary and secondary outcomes, which will be collected only in the follow up moments, aim at evaluating the impact of interventions. Considering that interventions are expected to influence either health and behavioral aspects, impact indicators are expected to provide information on changes in their frequency, and potential associations. On the other hand, data on the process indicators, which will be collected throughout the study, aim at assisting the evaluation of the quality and success of intervention’s delivery process. Thus, data regarding the provision, utilization and coverage aspects of the delivery process are expected to facilitate attributing the changes in primary and secondary outcomes to the intervention, by demonstrating that the intervention was of sufficient magnitude and occurred in a temporal sequence. Specifically, (1) provision indicators are expected to inform about the quality of provider’s training and performance, and if the requirements for provider’s optimal performance was delivered to them; (2) utilization indicators are expected to inform about the quality of receiver’s sensibilization, their receptivity to interventional activities, acquired knowledge and alterations of inadequate behaviors toward children; and (3) coverage indicators aim at providing information regarding the quality of the interventional activities, such as the adherence to them.

## 5. Statistical Analysis

### 5.1. Data Management

Questionnaire data, both from the three evaluation moments and from the six educational visits, will be double entered by CISA’s data loggers into digital forms forming an Open Data Kit (ODK) aggregate server. Afterwards, these files will be converted into Excel sheets and exported to SPSS for statistical analysis. Anthropometric indices will be extracted from the Excel files produced and entered into a WHO Anthro software (version 3.2.2, WHO, Geneva, Switzerland ) to calculate z-scores and classify malnutrition. The laboratorial data will also be similarly entered into Excel® (Microsoft Corporation, Redmond, Washington, DC, USA) files and exported to IBM SPSS software, version 24 (IBM Corp, Armonk, NY, USA) for statistical analysis.

### 5.2. Baseline Assessment Eevaluations

The nutritional quality of children’s diets (24–36 months) will be determined in accordance with the Children’s Individual Dietary Diversity Score Indicator (IDDS) [69,70]. The prevalence of malnutrition in children will be calculated by WHO Anthro software (version 3.2.2). The chi-square test will be used to compare proportions, in addition to calculating the attributable fractions.

Pre-and post-intervention and intervention versus control arms evaluations.

Primary and secondary impact indicators will be evaluated between the baseline and the follow ups (for each study sample), and between the intervention and control arms. Outcomes will be compared analyzing for variance in the continuous variables and logistical regression for the categorical variables. Adequacy and plausibility arguments will be used, by analyzing the process indicators (described in Table 2) in accordance with the conceptual maps. Relative risk will be calculated in order to compare the cumulative incidence of anemia among the intervention and control arms.

## 6. Discussion

The efficacy of interventions to reduce anemia can comprise both educational and therapeutic dimensions [2].

Deworming plus nutrition education has been reported to reduce anemia from 82.0% to 55.4%, while increasing the consumption of leafy green vegetables from 44.7% to 60.6% [24]. Furthermore, exclusive educational interventions targeting nutrition improvement, increased the ability of mothers to identify malnutrition (from 15% to 99%), increased exclusive breastfeeding (79% versus 48% in the control group), translated into weight gain (0.86kg versus 0.77kg in the control group), increased vegetable feeding, nutrient-dense foods at lunch (11% difference between the intervention and control groups), dietary requirements for energy, iron and zinc, complementary feeding, and significantly reduced rates of stunting [17,18,19,20,21,35,71].

Exclusive WASH educational interventions, health education on soil-transmitted helminths and schistosomiasis, significantly increased knowledge, reduced both the prevalence of *Ascaris lumbricoides* and *Schistosoma mansoni* and the incidence of hookworms and the intensity of trichuriasis, ascaridiasis and hookworms [25,26]. When deworming is added to WASH education, the prevalence and intensity of STH decrease and school children scored higher than the control on STH knowledge [26,31]. Studies evaluating educational approaches to prevent malaria report improved knowledge regarding breeding sites, bednet use and indoor spraying, increased windows and door net usage and alongside maintaining clean environment practices and leading to a reduced number of reported malaria episodes and the incidence of fever [27,28,39,72].

There are reports of intensity reduction (for *Schistosoma haematobium, Ascaris lumbricoides*, *Trichuris trichiura* and hookworm) and variable cure rates when participants are exposed only to deworming with PZQ or ALB. However, re-infections can occur rapidly [73,74,75,76,77,78,79,80,81,82]. While some authors report that the link between exclusive drug therapy and hemoglobin increase remains inconclusive, others report that exclusively deworming (with ALB and PZQ) may increase mean hemoglobin [83,84]. Additionally, treating malaria has an important impact on anemia reduction [85].

The studies investigating different combinations of those interventions present considerable differences in their designs and methodologies, making it hard to value and compare results [17,18,19,20,21,22,23,24,25,26,27,28,29,31,83,84,86,87,88]. Despite those results, only a few studies have simultaneously implemented a therapeutic plus educational mother-to-child nutrition intervention and compared it with a therapeutic plus educational mother-to-child WASH/Malaria practices [89].

Evaluating the efficacy of educational interventions represents a great challenge, as they are complex, have long causal pathways between the intervention and the outcome, span several intermediate levels and interacting components (in turn susceptible of modifying the effect under measurement and both the internal and external validity of the findings) [90]. One relevant dimension of this complexity is the number of target groups and the number of behaviors required by either those delivering or receiving the interventions for the impact on the outcomes to occur. Educational interventions depend on the knowledge acquired from field technicians, their performance in transmitting that knowledge, skills to conduct community activities and to monitor mother/caretaker practices. Additionally, mothers are expected to be receptive to counselling, gaining awareness of correct parental practices and changing inadequate behaviors and practices. In this study, we postulate that, for a single population, evaluation design, indicators, therapeutic approach and timeline, we will be able to more consistently and realistically describe and compare the effect of: 1) a therapeutic plus educational nutrition; and 2) a therapeutic plus WASH/Malaria interventions on the occurrence of anemia.

Nevertheless, considering the complexity of the design proposed, we can anticipate some difficulties. Randomization may not grant the elimination of all non-measured confounding factors just as the prevalence of several outcomes may be difficult to dilute within the clusters [91]. Additionally, there is also the possibility of poor compliance and differences in the intervention dosage delivered and the amount of response produced. Here, we will distribute nutrition and WASH/Malaria kits during the evaluation moments to prevent low adherence. Furthermore, we are also going to conduct an intensive training/retraining and performance evaluation system for providers, as well as a rotation strategy between technicians and families. The monitoring file of the receiver is expected to collect information on intermediate potential causal steps that will facilitate attributing the observed alterations in outcomes to the intervention, i.e., demonstrating that the intervention was of sufficient magnitude and occurred in a temporal sequence consistent with the hypothesized impact. Additionally, the results from complex cluster-randomized controlled trials, due to the stringencies of the probability statements required, may not be generalizable and further effectivity studies may be needed. Recommendations for overcoming these difficulties include recourse to adequacy and plausibility arguments, combining different types of evidence, in turn limiting the occurrence of bias, confounding factors, and chance [90]. In this study, detailed impact, provision, utilization and coverage indicators will be collected, as described in Table 2.

## 7. Conclusions

Exclusive therapeutic strategies have important results in decreasing prevalence and intensity of infections, however, in a contaminated environment, reinfections rapidly occur. In one hand, WASH/Malaria community-based educational approaches may sustain the achievements of therapeutics by decreasing transmission and, on the other hand, a more accurate evaluation of the effect of community-based educational strategies in adequate IYCF may occur when the influence of infections is removed. The design of this study aims at investigating the effect of 1) exclusive therapeutics (in a focused and context-adapted test-and-treat approach), 2) therapeutics plus educational WASH/Malaria and 3) therapeutics plus educational nutrition strategies. This will allow to clarify key questions that could help improving the control strategies targeting infectious-related and nutrition-related anemia in the country.

## Figures and Tables

**Table 1:** Summary of the treatment given in each arm.

Type of Action	Nutrition Arm:	WASH Arm:	Control Arm
Diagnosis and treatment of: - Malaria (artemether-Lumefantrine 20/120), - Schistosomiasis (Praziquantel) - Intestinal parasites (Albendazol and Tinidazol) Implemented at the baseline and then every 6 months until the end of the study.	Provided	Provided	Provided
Distribution of bednets at the baseline	Provided	Provided	Provided
Distribution of soap and lye at the first and second follow ups	Provided	Provided	Provided
Personalized, home-based counseling of primary caretakers	Provided	Provided	Not provided
Targets within the educational package Each visit will address 2 behaviors of the specified targets topics, moving to the next pair in later visits	(1) Breastfeeding; (2) Complementary feeding *; (3) Weekly adequate food diversity; (4) Appropriate number of meals; (5) Adequate amount of food; (6) Responsible feeding; (7) Disease and alimentation; (8) Hygiene and food safety.	(1) Bednet usage; (2) Reducing mosquito breeding sites (3) Prevention of open sky defecation; (4) Latrine cleaning; (5) Adequate hand washing; (6) Healthy backyard environment; (7) Adequate: water availability, treatment, transportation and storage; (8) Adequate personal hygiene.	Not applicable
Number of counselling visits	6	6	Not applicable
Activities within the community groups	(1) Discussion of nutritional value of foods, the effect of processing and the most common foodborne parasites; (2) Demonstration of fish-and-meat dishes; (3) Demonstration of combinations for breakfast and lunch; (4) Taste and score of recipes; (5) Distribution of illustrative notebook with recipes.	(1) Identification of focus of Soil-transmitted helminth (STH) contamination; (2) Identification of children’s risk behavior in the community; (3) Identification of household indoor resting mosquitos and outdoor breeding sites; (4) Construction of household washing hand mechanisms; (5) Correct montage of bednet; (6) Children hand-washing classes.	Not provided
Number of community group sessions	6	6	Not applicable
Total duration of the follow up	12 months	12 months	12 months

**Table 2:** Indicators of impact, provision, utilization and coverage being collected in this study.

Impact Evaluation	Process Evaluation		
	Provision * Indicators	Utilization Indicators	Coverage * Indicators
1. Primary outcomes 1.1 Impact on health - Variation of hemoglobin levels; - Anemia prevalence reduction. 2. Secondary outcomes 2.1 Impact on Health - Iron deficiency anaemia reduction; - Micronutrient deficiencies (iron, folate, vitamins A and B12, zinc and vitamin E serum levels); - Prevalence reduction, intensity, incidence and reinfections rates of STH, *P. falciparum* and *S. haematobium*; - Prevalence reduction of Stunting (height-for-age) and wasting (weight-for-height). 2.2 Impact on Behavior - Exclusive breastfeeding (0–5 months) and continued breastfeeding (12–15 months) [68]; - Increase of Minimum dietary diversity prevalence (6–23 months) [68]; - Increase of Minimum Meal Frequency prevalence [68]; - Increase of iron and vitamin A rich foods consumption; - Reduction of morbidity from diarrhea (reported).	1. Acquired knowledge - Theoretical test scores (evaluation of knowledge); - Practical test scores (evaluation of counseling performance); - Self-counseling efficacy evaluation. 2. Counseling performance - Number of families dropping outs from evaluation moments per technician; - Number of families successfully visited for counseling by each technician; - Number of notified participants present in each community groups per technician; - Number of scheduled visits held successfully per technician.	1. Acquired knowledge of receivers -Theoretical test scores; - Practical test scores (participation in community groups); - Self-evaluation at mother-to-child care. 2. Participatory level of receivers (adherence) - N° of attendances to evaluation moments; - N° of scheduled visits (both in person and by telephone call) held successfully; - N° of scheduled visits that had to be redials due to absence or unavailability; - Mood state of the receiver between visits; - N° of attendances to communitarian group. 3. Household environment evaluation (observation) - N° of household hand washing mechanisms; - N° of communitarian latrines around the house; - Cleaning conditions classification of household latrines (if existent); - Cleaning conditions classification of the backyard; - Presence of pets loose in the yard and stool. 4. Mother-to-children care practices - Children using bednets; - Children sidewalks; - Children with clean nails.	1. Services provided at evaluation moments - N° of hospital-based consultation held for parasite treatment; - N° of domiciliary-based consultation held for parasite treatment; - N° of hospital-based consultation held for sickle-cell follow up; - N° of children referred to the emergencies with severe anemia, severe malnutrition and non-malarial fever who reported to have been followed. 2.Participatory level of receivers (adherence) - Proportion of child-caretaker pair present in all evaluation moments; - Proportion of child-caretaker pair presenting in all counseling moments; - Proportion of caretakers participating in all community groups; - N° of notified child-mother pair failing the hospital-based consultations for parasite treatment or sickle-cell follow up; - N° of child-mother pair failing to picking up the sickle-cell drugs.

* Provision refers to the intervention provided by technicians and utilization refers to the action expected from receivers when provision is delivered.

## WHO Trial Registration Data Set

Primary registry and trial identifier number: ISRCTN: 18101157 (https://doi.org/10.1186/ISRCTN18101157); Date of primary registration: 11/04/2016; Source of monetary or material support: Calouste Gulbenkian Foundation, Banco de Fomento Angola, Special Program for Research and Training in Tropical Diseases and José Eduardo dos Santos Foundation; Primary sponsor: Health Research Center of Angola; Contact for public queries: claudia.videira@cisacaxito.org;

Public title: Anemia and its preventable etiologic agents in pre-school children from Bengo, Angola;

Scientific title: Efficacy of community educational interventions in nutrition and WASH/Malaria in reducing anemia in children under five, in the municipality of Dande—Angola;

Health conditions studied: Anemia and its etiologic agents;

Interventions: (1) Nutrition educational intervention plus screening and treatment of malaria, schistosomiasis and STH, (2) WASH and malaria educational intervention plus screening and treatment of malaria, schistosomiasis and STH and (3) exclusive screening and treatment of malaria, schistosomiasis and STH at the evaluation moments;

Key inclusion criteria: All children aged under 4, resident in hamlets with functional health posts;

Key exclusion criteria: Reported adverse reactions to albendazole and praziquantel and failing the baseline assessment and treatment;

Study type: Prevention;

Date of first enrolment: 15/06/2015;

Predicted date of the end of the study: 15/06/2019;

Target sample size: All children resident in hamlets with functional health posts will be considered eligible. Furthermore, there were 13 hamlets registered within the study area as having health posts, in which a density of 974 children was estimated using the CISA HDSS database; however, the functionality of those health posts needs further assessment in order to determine the clusters to be included and consequently the real sample size;

Recruitment status: No longer recruiting;

Primary outcome: Variation of hemoglobin levels between baseline, 6 and 12 months;

Key secondary outcomes: Variation of anthropometric indices, micronutrient deficiency and food diversity between baseline, 6 and 12 months.

## Abbreviations

ALB	Albendazole
CISA	Health Research Center of Angola (translated)
FPA	Food Photography Atlas
G6PD	Glucose Phosphate Dehydrogenase
HDSS	Health, Demographic and Surveillance System
HPLC	High-performance Liquid Chromatography
ID	Iron Deficiency
IDDS	Individual Dietary Diversity Score Indicator
IYCF	Infant and Young Child Feeding
PCR	Polymerase Chain Reaction
PZQ	Praziquantel
RCT	Randomized Controlled Trial
STH	Soil-Transmitted Helminth
UNICEF	United Nations Children’s Fund
WASH	Water, Sanitation and hygiene
WHO	World Health Organization

## References

[1] Brabin B.J., Premji Z., Verhoeff F. (2001). An analysis of anemia and child mortality. J. Nutr..

[2] Balarajan Y., Ramakrishnan U., Ozaltin E., Shankar A.H., Subramanian S.V. (2011). Anaemia in low-income and middle-income countries. Lancet.

[3] Guerrant D.I., Moore S.R., Lima A.A., Patrick P.D., Schorling J.B., Guerrant R.L. (1999). Association of early childhood diarrhea and cryptosporidiosis with impaired physical fitness and cognitive function four-seven years later in a poor urban community in northeast Brazil. Am. J. Trop. Med. Hyg..

[4] Halliday K.E., Karanja P., Turner E.L., Okello G., Njagi K., Dubeck M.M., Allen E., Jukes M.C., Brooker S.J. (2012). Plasmodium falciparum, anaemia and cognitive and educational performance among school children in an area of moderate malaria transmission: Baseline results of a cluster randomized trial on the coast of Kenya. Trop. Med. Int. Health.

[5] Sousa-Figueiredo J.C., Gamboa D., Pedro J.M., Fancony C., Langa A.J., Magalhaes R.J., Stothard J.R., Nery S.V. (2012). Epidemiology of malaria, schistosomiasis, geohelminths, anemia and malnutrition in the context of a demographic surveillance system in northern Angola. PLoS ONE.

[6] Soares Magalhaes R.J., Langa A., Pedro J.M., Sousa-Figueiredo J.C., Clements A.C., Vaz Nery S. (2013). Role of malnutrition and parasite infections in the spatial variation in children’s anaemia risk in northern Angola. Geospat. Health.

[7] WHO (2001). Iron Deficiency Anaemia Assessment, Prevention and Control a Guide for Programme Managers.

[8] Harding K.B., Neufeld L.M. (2012). Iron deficiency and anemia control for infants and young children in malaria-endemic areas: A call to action and consensus among the research community. Adv. Nutr..

[9] Sazawal S., Black R.E., Ramsan M., Chwaya H.M., Stoltzfus R.J., Dutta A., Dhingra U., Kabole I., Deb S., Othman M.K. (2006). Effects of routine prophylactic supplementation with iron and folic acid on admission to hospital and mortality in preschool children in a high malaria transmission setting: Community-based, randomised, placebo-controlled trial. Lancet.

[10] Clark M.A., Goheen M.M., Cerami C. (2014). Influence of host iron status on Plasmodium falciparum infection. Front. Pharm..

[11] Veenemans J., Milligan P., Prentice A.M., Schouten L.R., Inja N., van der Heijden A.C., de Boer L.C., Jansen E.J., Koopmans A.E., Enthoven W.T. (2011). Effect of supplementation with zinc and other micronutrients on malaria in Tanzanian children: A randomised trial. PLoS Med..

[12] Pasricha S.R., Drakesmith H., Black J., Hipgrave D., Biggs B.A. (2013). Control of iron deficiency anemia in low- and middle-income countries. Blood.

[13] Dreyfuss M.L., Stoltzfus R.J., Shrestha J.B., Pradhan E.K., LeClerq S.C., Khatry S.K., Shrestha S.R., Katz J., Albonico M., West K.P. (2000). Hookworms, malaria and vitamin A deficiency contribute to anemia and iron deficiency among pregnant women in the plains of Nepal. J. Nutr..

[14] Al-Mekhlafi H.M., Azlin M., Aini U.N., Shaik A., Sa’iah A., Fatmah M.S., Ismail M.G., Ahmad F., Aisah M.Y., Rozlida A.R. (2005). Protein-energy malnutrition and soil-transmitted helminthiases among Orang Asli children in Selangor, Malaysia. Asia Pac. J. Clin. Nutr..

[15] Suzuki T. (2016). Iron deficiency anemia refractory to iron preparations. Rinsho Ketsueki.

[16] WHO (2008). Worldwide Prevalence of Anaemia 1993–2005. WHO Global Database on Anaemia.

[17] Bhandari N., Bahl R., Mazumdar S., Martines J., Black R.E., Bhan M.K. (2003). Effect of community-based promotion of exclusive breastfeeding on diarrhoeal illness and growth: A cluster randomised controlled trial. Lancet.

[18] Aboud F.E., Moore A.C., Akhter S. (2008). Effectiveness of a community-based responsive feeding programme in rural Bangladesh: A cluster randomized field trial. Matern. Child Nutr..

[19] Roy S.K., Fuchs G.J., Mahmud Z., Ara G., Islam S., Shafique S., Akter S.S., Chakraborty B. (2005). Intensive nutrition education with or without supplementary feeding improves the nutritional status of moderately-malnourished children in Bangladesh. J. HealthPopul. Nutr..

[20] Roy S.K., Jolly S.P., Shafique S., Fuchs G.J., Mahmud Z., Chakraborty B., Roy S. (2007). Prevention of malnutrition among young children in rural Bangladesh by a food-health-care educational intervention: A randomized, controlled trial. Food Nutr. Bull..

[21] Lassi Z.S., Das J.K., Zahid G., Imdad A., Bhutta Z.A. (2013). Impact of education and provision of complementary feeding on growth and morbidity in children less than 2 years of age in developing countries: A systematic review. BMC Public Health.

[22] Imdad A., Yakoob M.Y., Bhutta Z.A. (2011). Impact of maternal education about complementary feeding and provision of complementary foods on child growth in developing countries. Bmc Public Health.

[23] Agbozo F., Colecraft E., Ellahi B. (2016). Impact of type of child growth intervention program on caregivers’ child feeding knowledge and practices: A comparative study in Ga West Municipality, Ghana. Food Sci. Nutr..

[24] Rao S., Joshi S., Bhide P., Puranik B., Asawari K. (2014). Dietary diversification for prevention of anaemia among women of childbearing age from rural India. Public Health Nutr..

[25] Al-Delaimy A.K., Al-Mekhlafi H.M., Lim Y.A., Nasr N.A., Sady H., Atroosh W.M., Mahmud R. (2014). Developing and evaluating health education learning package (HELP) to control soil-transmitted helminth infections among Orang Asli children in Malaysia. Parasites Vectors.

[26] Asaolu S.O., Ofoezie I.E. (2003). The role of health education and sanitation in the control of helminth infections. Acta Trop..

[27] Alvarado B.E., Gomez E., Serra M., Carvajal R., Carrasquilla G. (2006). Evaluation of an educational strategy on malaria in rural areas of the Colombian Pacific Coast. Biomedica.

[28] Amoran O.E. (2013). Impact of health education intervention on malaria prevention practices among nursing mothers in rural communities in Nigeria. Niger. Med J. J. Niger. Med. Assoc..

[29] Lee Y.H., Jeong H.G., Kong W.H., Lee S.H., Cho H.I., Nam H.S., Ismail H.A., Alla G.N., Oh C.H., Hong S.T. (2015). Reduction of urogenital schistosomiasis with an integrated control project in Sudan. PLoS Negl. Trop. Dis..

[30] Foote E.M., Sullivan K.M., Ruth L.J., Oremo J., Sadumah I., Williams T.N., Suchdev P.S. (2013). Determinants of anemia among preschool children in rural, western Kenya. Am. J. Trop. Med. Hyg..

[31] Gyorkos T.W., Maheu-Giroux M., Blouin B., Casapia M. (2013). Impact of health education on soil-transmitted helminth infections in schoolchildren of the Peruvian Amazon: A cluster-randomized controlled trial. PLoS Negl. Trop. Dis..

[32] Allen L.H. (2008). To what extent can food-based approaches improve micronutrient status?. Asia Pac. J. Clin. Nutr..

[33] Humphrey J.H., Mbuya M.N.N., Ntozini R., Moulton L.H., Stoltzfus R.J., Tavengwa N.V., Mutasa K., Majo F., Mutasa B., Mangwadu G. (2019). Independent and combined effects of improved water, sanitation, and hygiene, and improved complementary feeding, on child stunting and anaemia in rural Zimbabwe: A cluster-randomised trial. Lancet Glob Health.

[34] Null C., Stewart C.P., Pickering A.J., Dentz H.N., Arnold B.F., Arnold C.D., Benjamin-Chung J., Clasen T., Dewey K.G., Fernald L.C.H. (2018). Effects of water quality, sanitation, handwashing, and nutritional interventions on diarrhoea and child growth in rural Kenya: A cluster-randomised controlled trial. Lancet Glob. Health.

[35] Penny M.E., Creed-Kanashiro H.M., Robert R.C., Narro M.R., Caulfield L.E., Black R.E. (2005). Effectiveness of an educational intervention delivered through the health services to improve nutrition in young children: A cluster-randomised controlled trial. Lancet.

[36] Diamond J.J. (2007). Development of a reliable and construct valid measure of nutritional literacy in adults. Nutr. J..

[37] Nasr N.A., Al-Mekhlafi H.M., Ahmed A., Roslan M.A., Bulgiba A. (2013). Towards an effective control programme of soil-transmitted helminth infections among Orang Asli in rural Malaysia. Part 1: Prevalence and associated key factors. Parasites Vectors.

[38] Schmidlin T., Hurlimann E., Silue K.D., Yapi R.B., Houngbedji C., Kouadio B.A., Acka-Douabele C.A., Kouassi D., Ouattara M., Zouzou F. (2013). Effects of hygiene and defecation behavior on helminths and intestinal protozoa infections in Taabo, Cote d’Ivoire. PLoS ONE.

[39] Tobgay T., Pem D., Dophu U., Dumre S.P., Na-Bangchang K., Torres C.E. (2013). Community-directed educational intervention for malaria elimination in Bhutan: Quasi-experimental study in malaria endemic areas of Sarpang district. Malar. J..

[40] Chirdan O.O., Zoakah A.I., Ejembi C.L. (2008). Impact of health education on home treatment and prevention of malaria in Jengre, North Central Nigeria. Ann. Afr. Med..

[41] Pasricha S.R., Vijaykumar V., Prashanth N.S., Sudarshan H., Biggs B.A., Black J., Shet A. (2009). A community based field research project investigating anaemia amongst young children living in rural Karnataka, India: A cross sectional study. BMC Public Health.

[42] Rosario E.V.N., Costa D., Francisco D., Brito M. (2017). HDSS Profile: The Dande Health and Demographic Surveillance System (Dande HDSS, Angola). Int. J. Epidemiol..

[43] MINSA (2014). Plano Municipal de Desenvolvimento Sanitário, 2013–2017.

[44] ProPAN (2013). ProPAN: Process for the Promotion of Child Feeding. Software User’s Guide Version 2.0..

[45] WHO (2009). WHO Child Growth Standards: Growth Velocity Based on Weight, Length and Head Circumference: Methods and Development.

[46] Yang H., de Onis M. (2008). Algorithms for converting estimates of child malnutrition based on the NCHS reference into estimates based on the WHO Child Growth Standards. BMC Pediatr..

[47] WHO (2010). WHO Guidelines on Drawing Blood: Best Practices in Phlebotomy.

[48] Katz N., Chaves A., Pellegrino J. (1972). A simple device for quantitative stool thick-smear technique in Schistosomiasis mansoni. Rev. Inst. Med. Trop. Sao Paulo.

[49] WHO (1991). Basic Laboratory Methods in Medical Parasitology.

[50] Vitros. https://www.cmmc.org/cmmclab/IFU/Fol_GEM1355_WW_EN_I.pdf.

[51] Ferritin Q. http://www.qca.es/en/exp/prod/ferritin-85.

[52] Zhao B., Tham S.Y., Lu J., Lai M.H., Lee L.K., Moochhala S.M. (2004). Simultaneous determination of vitamins C, E and beta-carotene in human plasma by high-performance liquid chromatography with photodiode-array detection. J. Pharm. Pharm. Sci..

[53] Zinc Q. http://www.qca.es/en/exp/prod/zinc-215.

[54] Brito M.T.C., Santos B., Veiga L. (2014). Glucose-6-Phosphate Dehydrogenase Deficiency in Children from 0 to 14 Years Hospitalized at the Pediatric Hospital David Bernardino, Luanda, Angola. J. Pharmacogenom. Pharmacoproteom..

[55] Waterfall C.M., Cobb B.D. (2001). Single tube genotyping of sickle cell anaemia using PCR-based SNP analysis. Nucleic Acids Res..

[56] WHO (2015). Guidlines for the Treatment of Malaria.

[57] Stothard J.R., Sousa-Figueiredo J.C., Betson M., Green H.K., Seto E.Y., Garba A., Sacko M., Mutapi F., Vaz Nery S., Amin M.A. (2011). Closing the praziquantel treatment gap: New steps in epidemiological monitoring and control of schistosomiasis in African infants and preschool-aged children. Parasitology.

[58] WHO (2006). Preventive Chemotherapy in Human Helminthiasi: Coordinated Use of Anthelminthic Drugs in Control Interventions: A Manual for Health Professionals and Programme Managers.

[59] WHO (2010). Report of a Meeting to Review the Results of Studies on the Treatment of Schistosomiasis in Preschool-Age Children.

[60] WHO (2013). Essential Nutrition Actions: Improving Maternal, Newborn, Infant and Young Child Health and Nutrition.

[61] WHO (2015). Improving Nutrition Outcomes with Better Water, Sanitation and Hygiene: Practical Solutions for Policies and Programmes.

[62] WHO (2010). Indicators for Assessing Infant and Young Child Feeding Practices Part 2: Measurement.

[63] Moshe G., Amitai Y., Korchia G., Korchia L., Tenenbaum A., Rosenblum J., Schechter A. (2013). Anemia and iron deficiency in children: Association with red meat and poultry consumption. J. Pediatr. Gastroenterol. Nutr..

[64] Davidsson L., Haskell M. (2011). Bioavailability of micronutrients: Stable isotope techniques to develop effective food-based strategies to combat micronutrient deficiencies. Food Nutr. Bull..

[65] De la Cruz-Gongora V., Villalpando S., Shamah-Levy T. (2018). Prevalence of anemia and consumption of iron-rich food groups in Mexican children and adolescents: Ensanut MC 2016. Salud Publica Mex..

[66] DeFries R., Chhatre A., Davis K.F., Dutta A., Fanzo J., Ghosh-Jerath S., Myers S., Rao N.D., Smith M.R. (2018). Impact of Historical Changes in Coarse Cereals Consumption in India on Micronutrient Intake and Anemia Prevalence. Food Nutr. Bull..

[67] Eussen S., Alles M., Uijterschout L., Brus F., van der Horst-Graat J. (2015). Iron intake and status of children aged 6-36 months in Europe: A systematic review. Ann. Nutr. Metab..

[68] WHO (2007). Indicators for Assessing Infant and Young Child Feeding Practices: Conclusions of a Consensus. Meeting Held 6–8 November 2007 in Washington, DC, USA..

[69] Hoddinott J.a.Y.Y. (2002). Dietary Diversity as a Household Food Security Indicator.

[70] Kennedy G., Ballard T., Dop M.C. (2011). Guidelines for Measuring Household and Individual Dietary Diversity.

[71] Coutinho S.B., de Lira P.I., de Carvalho Lima M., Ashworth A. (2005). Comparison of the effect of two systems for the promotion of exclusive breastfeeding. Lancet.

[72] Abegunde D., Orobaton N., Bassi A., Oguntunde O., Bamidele M., Abdulkrim M., Nwizugbe E. (2016). The Impact of Integrated Community Case Management of Childhood Diseases Interventions to Prevent Malaria Fever in Children Less than Five Years Old in Bauchi State of Nigeria. PLoS ONE.

[73] Narain K., Medhi G.K., Rajguru S.K., Mahanta J. (2004). Cure and reinfection patterns of geohelminthic infections after treatment in communities inhabiting the tropical rainforest of Assam, India. Southeast Asian J. Trop. Med. Public Health.

[74] Yap P., Du Z.W., Wu F.W., Jiang J.Y., Chen R., Zhou X.N., Hattendorf J., Utzinger J., Steinmann P. (2013). Rapid re-infection with soil-transmitted helminths after triple-dose albendazole treatment of school-aged children in Yunnan, People’s Republic of China. Am. J. Trop. Med. Hyg..

[75] Keiser J., Utzinger J. (2008). Efficacy of current drugs against soil-transmitted helminth infections: Systematic review and meta-analysis. JAMA J. Am. Med. Assoc..

[76] Soukhathammavong P.A., Sayasone S., Phongluxa K., Xayaseng V., Utzinger J., Vounatsou P., Hatz C., Akkhavong K., Keiser J., Odermatt P. (2012). Low efficacy of single-dose albendazole and mebendazole against hookworm and effect on concomitant helminth infection in Lao PDR. PLoS Negl. Trop. Dis..

[77] Tchuem Tchuente L.A., Momo S.C., Stothard J.R., Rollinson D. (2013). Efficacy of praziquantel and reinfection patterns in single and mixed infection foci for intestinal and urogenital schistosomiasis in Cameroon. Acta Trop..

[78] Tchuente L.A., Shaw D.J., Polla L., Cioli D., Vercruysse J. (2004). Efficacy of praziquantel against Schistosoma haematobium infection in children. Am. J. Trop. Med. Hyg..

[79] Midzi N., Sangweme D., Zinyowera S., Mapingure M.P., Brouwer K.C., Kumar N., Mutapi F., Woelk G., Mduluza T. (2008). Efficacy and side effects of praziquantel treatment against *Schistosoma haematobium* infection among primary school children in Zimbabwe. Trans. R. Soc. Trop. Med. Hyg..

[80] Saathoff E., Olsen A., Magnussen P., Kvalsvig J.D., Becker W., Appleton C.C. (2004). Patterns of Schistosoma haematobium infection, impact of praziquantel treatment and re-infection after treatment in a cohort of schoolchildren from rural KwaZulu-Natal/South Africa. BMC Infect. Dis..

[81] Webster B.L., Diaw O.T., Seye M.M., Faye D.S., Stothard J.R., Sousa-Figueiredo J.C., Rollinson D. (2013). Praziquantel treatment of school children from single and mixed infection foci of intestinal and urogenital schistosomiasis along the Senegal River Basin: Monitoring treatment success and re-infection patterns. Acta Trop..

[82] Wang W., Wang L., Liang Y.S. (2012). Susceptibility or resistance of praziquantel in human schistosomiasis: A review. Parasitol. Res..

[83] Kinung’hi S.M., Magnussen P., Kishamawe C., Todd J., Vennervald B.J. (2015). The impact of anthelmintic treatment intervention on malaria infection and anaemia in school and preschool children in Magu district, Tanzania: An open label randomised intervention trial. BMC Infect. Dis..

[84] Taylor-Robinson D.C., Maayan N., Soares-Weiser K., Donegan S., Garner P. (2015). Deworming drugs for soil-transmitted intestinal worms in children: Effects on nutritional indicators, haemoglobin, and school performance. Cochrane Database Syst. Rev..

[85] Guthmann J.P., Cohuet S., Rigutto C., Fortes F., Saraiva N., Kiguli J., Kyomuhendo J., Francis M., Noel F., Mulemba M. (2006). High efficacy of two artemisinin-based combinations (artesunate + amodiaquine and artemether + lumefantrine) in Caala, Central Angola. Am. J. Trop. Med. Hyg..

[86] Esrey S.A., Potash J.B., Roberts L., Shiff C. (1991). Effects of improved water supply and sanitation on ascariasis, diarrhoea, dracunculiasis, hookworm infection, schistosomiasis, and trachoma. Bull. World Health Organ..

[87] Amoran O.E., Fatugase K.O., Fatugase O.M., Alausa K.O. (2012). Impact of health education intervention on insecticide treated nets uptake among nursing mothers in rural communities in Nigeria. Bmc Res. Notes.

[88] Nery S.V., McCarthy J.S., Traub R., Andrews R.M., Black J., Gray D., Weking E., Atkinson J.A., Campbell S., Francis N. (2015). A cluster-randomised controlled trial integrating a community-based water, sanitation and hygiene programme, with mass distribution of albendazole to reduce intestinal parasites in Timor-Leste: The WASH for WORMS research protocol. BMJ Open.

[89] Humphrey J.H., Jones A.D., Manges A., Mangwadu G., Maluccio J.A., Mbuya M.N., Moulton L.H., Ntozini R., Prendergast A.J., Sanitation Hygiene Infant Nutrition Efficacy (SHINE) Trial Team (2015). The Sanitation Hygiene Infant Nutrition Efficacy (SHINE) Trial: Rationale, Design, and Methods. Clin. Infect. Dis..

[90] Victora C.G., Habicht J.P., Bryce J. (2004). Evidence-based public health: Moving beyond randomized trials. Am. J. Public Health.

[91] Habicht J.P., Victora C.G., Vaughan J.P. (1999). Evaluation designs for adequacy, plausibility and probability of public health programme performance and impact. Int. J. Epidemiol..

